# A Novel *Shigella* O-Polysaccharide–IpaB Conjugate Vaccine Elicits Robust Antibody Responses and Confers Protection against Multiple *Shigella* Serotypes

**DOI:** 10.1128/msphere.00019-23

**Published:** 2023-04-05

**Authors:** Girmay Desalegn, Neeraj Kapoor, Lucy Pill-Pepe, Leslie Bautista, Lu Yin, Esther Ndungo, Edwin V. Oaks, Jeff Fairman, Marcela F. Pasetti

**Affiliations:** a Center for Vaccine Development and Global Health (CVD), University of Maryland School of Medicine, Baltimore, Maryland, USA; b Vaxcyte, Inc., San Carlos, California, USA; c Patuxent Research and Consulting Group, Gambrills, Maryland, USA; University of Florida

**Keywords:** *Shigella*, conjugate vaccine, O-polysaccharide (OPS), IpaB, antibody, protection

## Abstract

*Shigella* is responsible for high burdens of diarrhea and dysentery globally. Children living in areas of endemicity are the most affected, and currently, there are no licensed vaccines to prevent shigellosis. Vaccine approaches have traditionally targeted the bacterial lipopolysaccharide as a protective antigen. *Shigella* O-polysaccharide (OPS) conjugated to recombinant Pseudomonas aeruginosa exotoxin A (rEPA) or tetanus toxoid (TT) is advanced in clinical evaluation. Adequate efficacy of these vaccines, particularly in the infant target group, remains to be demonstrated. A major limitation of the OPS-glycoconjugate concept is its limited coverage, since immunity to the O antigen is serotype specific, and there are multiple disease-causing serotypes. Another concern is the use of protein carriers already included in multiple other childhood vaccines. This study reports a novel *Shigella* OPS conjugate vaccine that uses the *Shigella* invasion plasmid antigen B (IpaB) as the carrier protein. IpaB is a virulence factor component of the *Shigella* type III secretion system and highly conserved among *Shigella* serotypes. It is robustly immunogenic and a protective antigen. IpaB and IpaB containing nonnative amino acids (nnAA) were produced at large scale using cell-free protein synthesis. Incorporation of nnAA enabled site-specific conjugation of IpaB to Shigella flexneri 2a OPS using click chemistry, yielding OPS-IpaB glycoconjugate. Parenteral immunization of mice with the OPS-IpaB vaccine resulted in high levels of OPS- and IpaB-specific serum IgG and robust protection against lethal S. flexneri 2a or Shigella sonnei challenge. The OPS-IpaB vaccine is a promising new vaccine candidate with the capacity to confer broad protection against clinically relevant *Shigella* serotypes.

**IMPORTANCE** Diarrhea caused by *Shigella* species results in long-term disability and mortality globally, disproportionally affecting younger children living in poor countries. Although it is treatable by antibiotics, the rapid and widespread emergence of resistant strains and the highly contagious nature of the disease compel the development of preventive tools. Currently, several *Shigella* OPS conjugate vaccines are being evaluated in clinical studies, but these rely exclusively on immunity against the bacterial O antigen, which limits their coverage to only the immunizing serotype; multivalent vaccines are needed to protect against the most prevalent serotypes. This is the first report of a novel *Shigella* OPS-conjugate vaccine that uses *Shigella* IpaB as a carrier and protective antigen. This vaccine, administered parenterally, elicited robust immunity and protected mice against lethal infection by S. flexneri 2a or S. sonnei. The OPS-IpaB vaccine is a promising candidate for evaluation in vulnerable populations.

## INTRODUCTION

*Shigella* species are responsible for a high burden of moderate to severe diarrhea globally. Children younger than 5 years of age, particularly toddlers 2 to 3 years old, are the most affected (1–3). Repeated infection impairs physical and cognitive development, leading to lifelong disability (4). The rapid and widespread prevalence of antibiotic-resistant *Shigella* species has also become a major public health concern (5, 6). Currently, there are no approved vaccines against shigellosis. The bacterial O-polysaccharide (OPS) is an essential virulence factor and the main target antigen of most current vaccine candidates being tested in the clinic. Shigella flexneri 2a OPS or synthetic oligosaccharide conjugated to tetanus toxoid (TT) (7–9), mutant diphtheria toxoid cross-reacting material 197 (CRM_197_) (10, 11), or recombinant Pseudomonas aeruginosa exotoxin A (rEPA) (9–15) has been safe and immunogenic in humans. Flexyn2a, the bioconjugate of S. flexneri 2a OPS-rEPA, was moderately protective and only against severe disease in North American volunteers in a recent controlled human infection model (CHIM) study (12). An earlier Shigella sonnei OPS-rEPA conjugate was effective in adults and older children but not in toddlers, the primary vaccine target group (14, 15). A tetravalent bioconjugate OPS-rEPA formulation is being tested in 9-month-old infants and children 2 to 5 years of age living in Kenya (NCT04056117). SF2a-TT15, a synthetic S. flexneri 2a oligosaccharide conjugated to TT (7), is being evaluated for efficacy in an adult study at the University of Maryland (NCT04078022). The immunogenicity of this vaccine is also being investigated in Kenyan children 9 months of age and older (NCT04602975). Finally, a bivalent S. flexneri 2a and S. sonnei OPS-TT conjugate vaccine is being evaluated in a phase 3 efficacy study (NCT05156528).

A major limitation of these glycoconjugates is their exclusive reliance on anti-OPS immunity, which is specific for each *Shigella* serotype; a multivalent vaccine with at least 4 OPS conjugates would be needed to prevent infection caused by the most prevalent disease-causing serotypes (S. flexneri 2a, 3a, and 6, and S. sonnei). Here, we describe the development of a novel *Shigella* OPS-based conjugate vaccine that uses *Shigella* invasion plasmid antigen B (IpaB), a type III secretion system protein highly conserved among all *Shigella* serotypes (16), as the carrier protein. IpaB is a highly immunogenic and protective antigen in mice (17, 18). IpaB antibody levels (19) and their phagocytic activity involving innate immune cells (20) have been associated with clinical protection in experimentally infected human volunteers.

We have successfully modified the native *ipaB* gene to site-selectively incorporate nonnative amino acids (nnAA) with reactive azide side chains into the protein’s backbone and successfully produced high yields of the engineered protein using cell-free protein synthesis (CFPS). Using click-chemistry reactions, nnAA-containing IpaB was successfully conjugated to dibenzocyclooctyne (DBCO)-derivatized S. flexneri 2a core OPS (here referred to as OPS) to generate a novel conjugate vaccine, namely, OPS-IpaB. Immunization of mice with S. flexneri 2a OPS-IpaB elicited strong antibody responses and protected against lethal challenge with S. flexneri 2a or S. sonnei. Importantly, the protective efficacy of the OPS-IpaB conjugate was superior to that afforded by a conjugate vaccine consisting of S. flexneri 2a OPS conjugated to CRM_197_.

## RESULTS

### Generation of OPS-IpaB conjugate vaccine.

Our approach to producing a *Shigella* OPS-IpaB conjugate consisted of (i) acetic acid extraction of *Shigella* OPS, (ii) production of IpaB containing nnAA using CFPS, and (iii) conjugation of OPS to IpaB using click chemistry. S. flexneri 2a core OPS was extracted from a live attenuated S. flexneri 2a strain (CVD1204) (21) through acetic acid hydrolysis (see Materials and Methods). OPS extraction was efficient (>250 mg/L of culture, residual proteins below limit of detection, <0.1% nucleic acid, and <50 endotoxin units/mg endotoxin) and reproducible. Size exclusion chromatography combined with multiangle light scattering (SEC-MALS) analysis revealed a heterogeneous polysaccharide (PS) with an average molar mass of ~36 kDa (see Fig. S1A in the supplemental material). High-pH anion-exchange chromatography with pulsed amperometric detection (HPAEC-PAD) analysis confirmed that the purified OPS comprised rhamnose, *N*-acetylglucosamine, and glucose sugar moieties (Fig. S1B).

10.1128/msphere.00019-23.1 FIG S1 Characterization of purified S. flexneri 2a OPS. (A) SEC-MALS determination of molar mass and concentration (dRI) of native S. flexneri 2a core-OPS. SEC-MALS analysis showed a purified OPS with an estimated average molar mass of ~36 kDa. LS, light scattering; dRI, refractive index. (B) Representation of sugar composition in the purified OPS isolated from S. flexneri 2a through Dionex HPAEC-PAD analysis. Peaks represent rhamnose, *N*-acetylglucosamine, and glucose (left to right) sugar moieties in the OPS. Download FIG S1, TIF file, 1.5 MB. Copyright © 2023 Desalegn et al. 2023 Desalegn et al. https://creativecommons.org/licenses/by/4.0/ This content is distributed under the terms of the Creative Commons Attribution 4.0 International license.

**FIG 1:**
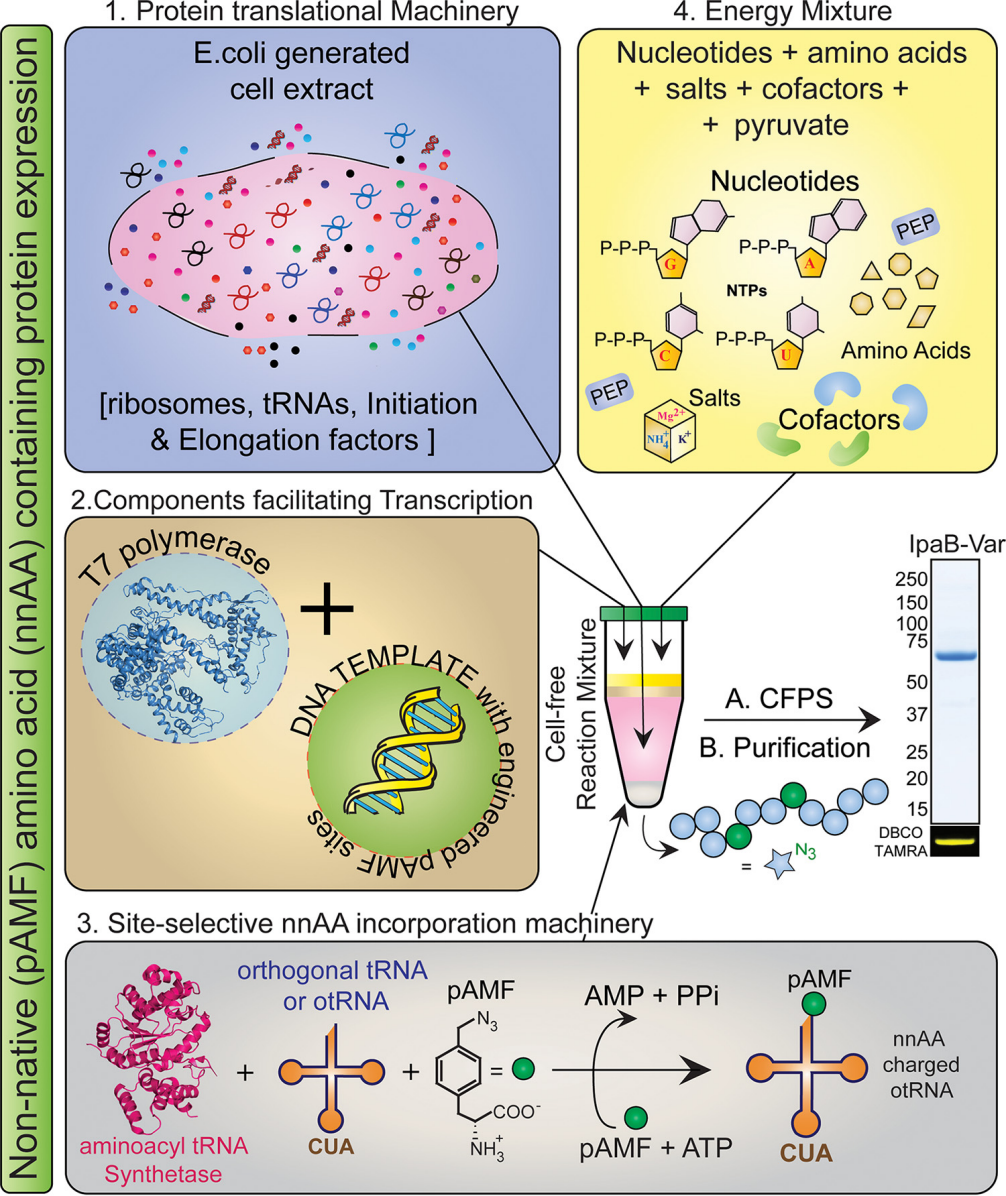
Schematic representation of the expression of nnAA-containing IpaB using XpressCF^+^ CFPS technology. Cell-free protein synthesis (CFPS) for expression of nnAA (pAMF) containing IpaB was performed by generating a reaction mixture comprising (1) Escherichia coli cell extract, (2) components necessary for transcription (T7 polymerase and the DNA template), and (3) molecular machinery facilitating site-selective pAMF incorporation (synthetase, orthogonal tRNA [otRNA], and pAMF) along with (4) essential components necessary for energy production (nucleoside triphosphates [NTPs], amino acids, salts, and cofactors). Postexpression, CFPS product was harvested, and target protein (IpaB-var) was purified and analyzed by SDS-PAGE analysis. pAMF incorporation was confirmed through labeling with DBCO-TAMRA dye. var, variant.

**FIG 2:**
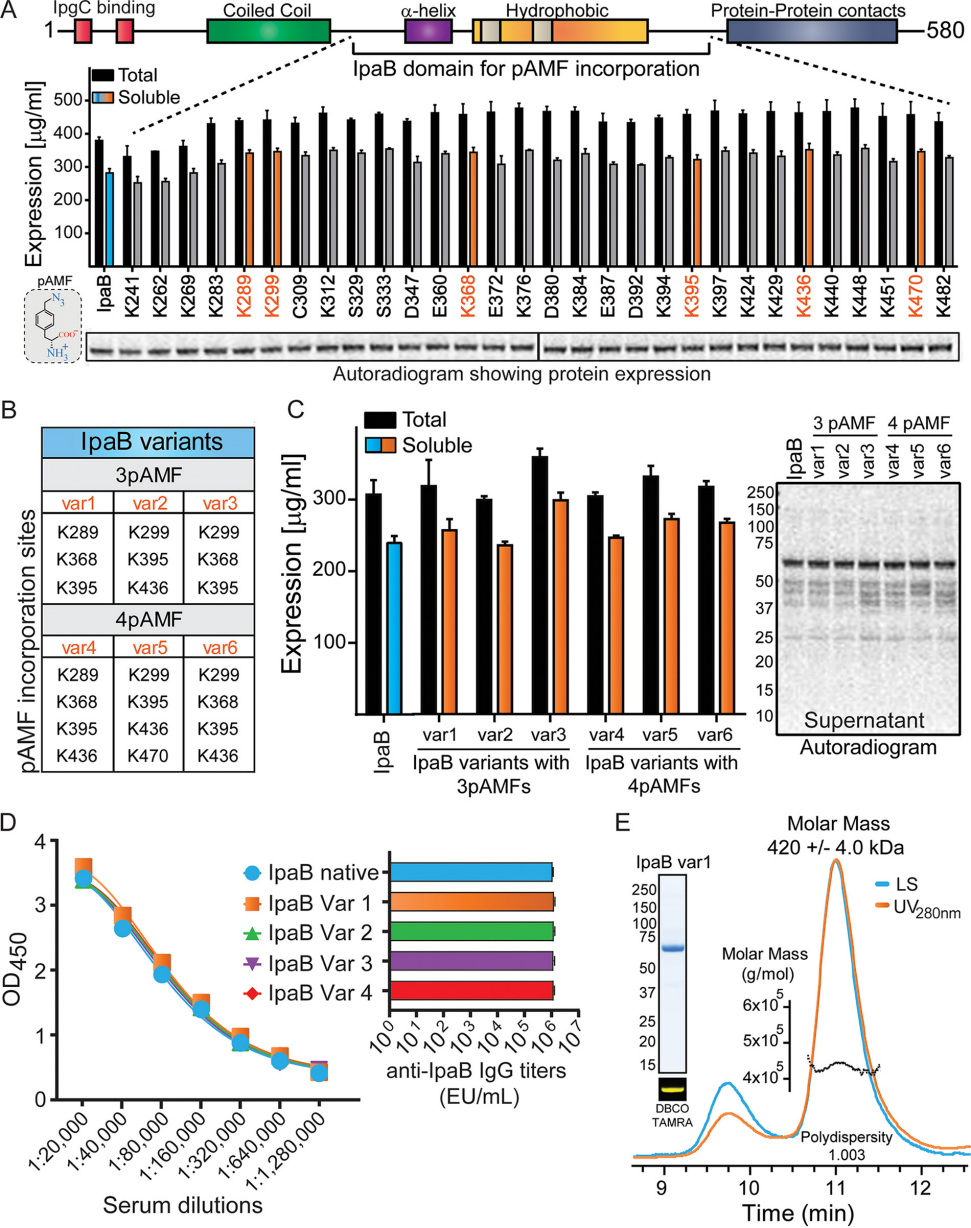
Design, expression, and biophysical characterization of nnAA-containing IpaB alone or after conjugation to OPS-DBCO. (A) Native sequence of full-length IpaB with modular architecture was utilized to generate single-site pAMF incorporation variants at the listed sites, spanning the core of the protein. Expression of native IpaB (blue) and each of the variants (gray/orange) was determined using [^14^C]leucine incorporation into the translating polypeptide followed by autoradiogram recording. (B) pAMF incorporation sites with minimal impact on protein expression were selected (orange) and combined to generate 3 pAMF (var1 to -3) or 4 pAMF (var4 to -6)-containing variants. (C) The expression of var1 to -6 relative to native IpaB was determined using [^14^C]leucine incorporation assay. Autoradiogram analysis of the recovered supernatants shows expression of all IpaB variants in comparison to native IpaB. (D) Four-parameter logistic (4PL) dose-response curves of absorbance values at 450 nm versus serum dilution factor (left panel), and ELISA serum IgG titers (right panel) comparing immune reactivities of native IpaB and IpaB variants using human *Shigella* convalescent-phase sera. Data represent mean titer (obtained from multiple dilutions tested) ± standard error of the mean; native IpaB versus IpaB variants, *P* > 0.74 by *t* test. (E) Safe-blue-stained SDS-PAGE analysis of purified IpaB-var1; selective labeling with DBCO-TAMRA fluorescent dye confirmed pAMF incorporation. SEC-MALS analysis of purified IpaB-var1 showed a monodisperse distribution with an estimated molar mass of 420 ± 4 kDa. var, variant.

**FIG 3:**
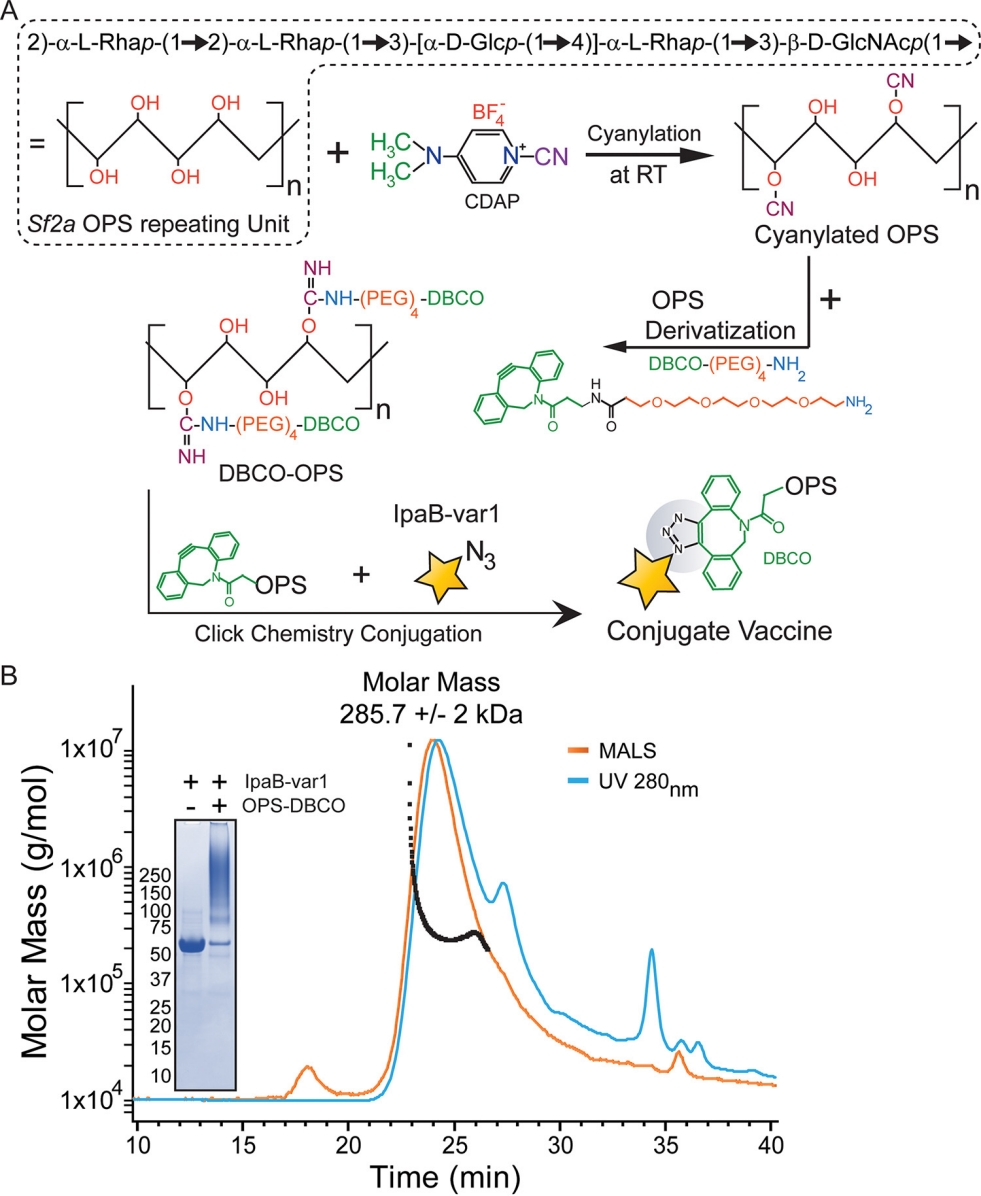
OPS-IpaB conjugation. (A) Schematic representation of OPS-IpaB conjugation process using IpaB-var1 and DBCO-derivatized OPS. Purified IpaB-var1 was incubated with S. flexneri 2a OPS-DBCO at room temperature (RT), and a click-chemistry reaction was performed to produce OPS-IpaB conjugate. (B) Safe-blue-stained SDS-PAGE analysis showing efficient conjugation of IpaB-var1 to OPS-DBCO. SEC-MALS analysis of the purified conjugates estimates an average molar mass of 285.7 ± 2 kDa for the OPS-IpaB conjugate. var, variant.

IpaB was produced using Vaxcyte’s proprietary Xpress^+^ CFPS platform (22). The native protein sequence was used as a template to generate multiple IpaB single-site variants that efficiently incorporated nnAA *p*-azidophenylalanine (pAMF) (Fig. 1), at specific sites purposely selected to be distant from immunologically relevant epitopes (23, 24). Expression yields of both native and nnAA-containing IpaB were >200 mg/L as measured through [^14^C]leucine incorporation (Fig. 2A). Six of the single-site IpaB pAMF incorporation variants (highlighted in orange in Fig. 2A) were selected based on expression levels, and their pAMF sites were combined to generate 3 and 4 pAMF-containing IpaB (variants 1 to 6) as indicated in Fig. 2B. Expression of IpaB by the 3 or 4 pAMF-containing variants compared to native IpaB was estimated using [^14^C]leucine incorporation followed by autoradiogram analysis and was found to be largely unaffected by pAMF incorporation (Fig. 2C). Importantly, the immune reactivity of IpaB variants 1 to 4 was compared to that of native IpaB using human *Shigella* convalescent-phase sera and was found to be almost identical (Fig. 2D), confirming the preservation of immune reactive epitopes. IpaB-variant 1 (IpaB-var1), which produced larger conjugates in initial SEC-MALS analysis (data not shown), was selected as the carrier protein, scaled up using CFPS (performed at 2- to 4-L reaction volume in a bioreactor under controlled conditions), and produced at high purity and homogeneity. Biophysical characterization of the purified protein using SEC-MALS estimated a molar mass of 420 ± 0.4 kDa (Fig. 2E), which is a close approximation to the mass of a hexamer of IpaB in solution (theoretical molecular mass of monomeric IpaB-variant 1, ~65 kDa). SDS-PAGE analysis of the purified protein revealed >95% purity, and the dibenzocyclooctyne-tetramethylrhodamine (DBCO-TAMRA) labeling confirmed the presence of pAMF sites (Fig. 2E, inset). We next performed DBCO derivatization of S. flexneri 2a OPS (OPS-DBCO) followed by click-chemistry reaction with IpaB-variant 1 to produce the OPS-IpaB conjugate vaccine (Fig. 3A). SEC-MALS analysis of OPS-IpaB revealed an average molecular mass of 285 ± 2 kDa (Fig. 3B), and SDS-PAGE analysis confirmed almost complete utilization of IpaB in the conjugation reaction (Fig. 3B, inset). In addition, OPS-DBCO was also conjugated to CFPS-produced eCRM, an nnAA-containing variant of diphtheria toxin variant CRM_197_ (25), to generate OPS-CRM for use as a control in mouse experiments.

### Immunogenicity of S. flexneri 2a OPS-IpaB conjugate vaccine.

**FIG 4:**
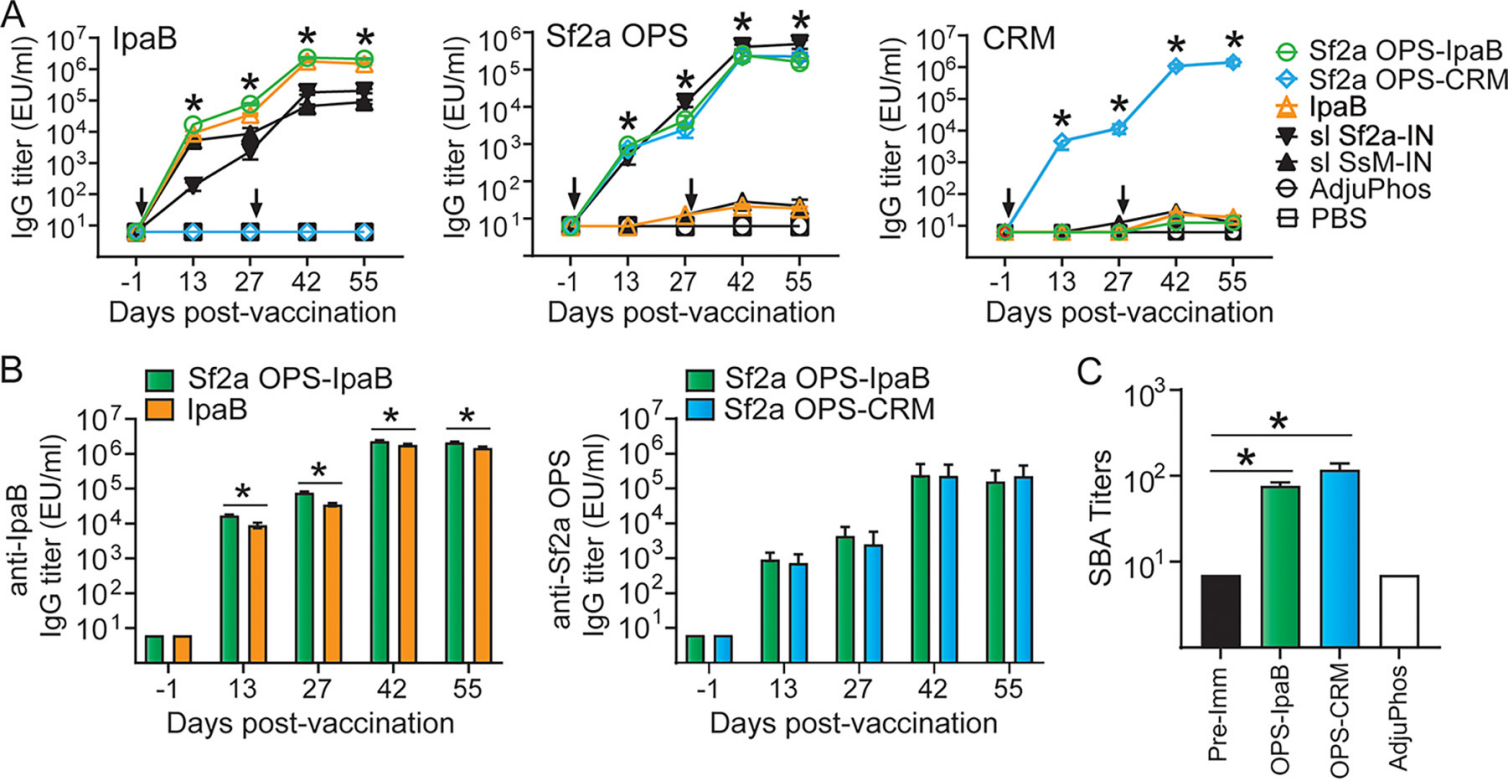
Immunogenicity of S. flexneri 2a OPS-IpaB conjugate vaccine. Mice (20/group/challenge strain) were immunized intramuscularly (i.m.) on days 0 and 28 with 10 μg of S. flexneri 2a OPS-IpaB, OPS-CRM, or IpaB, each admixed with AdjuPhos. Negative-control groups received PBS or AdjuPhos. Mice immunized intranasally (i.n.) with sublethal doses (10^5^ CFU) of S. flexneri 2a (Sf2a) or S. sonnei (SsM) were included as positive controls for S. flexneri 2a and S. sonnei challenge, respectively. (A) IpaB-, S. flexneri 2a OPS-, and CRM-specific serum IgG measured by ELISA; arrows indicate immunization. Mean of individual titers ± standard error of the mean; *, *P* < 0.001 versus AdjuPhos and PBS by *t* test. (B) IpaB- and OPS-IgG titer comparison for specific groups; *, *P* < 0.01 by *t* test. (C) S. flexneri 2a serum bactericidal activity (SBA) on day 55. Mean of individual titers ± standard error of the mean; *, *P* < 0.001 versus prevaccination by *t* test. sl Sf2a-IN, sublethal dose of S. flexneri 2a-IN; sl SsM-IN, sublethal dose of S. sonnei Moseley-IN.

BALB/c mice were immunized intramuscularly (i.m.) on two occasions (days 0 and 28) with 10 μg/dose of OPS-IpaB admixed with an alum-based adjuvant, AdjuPhos. Groups receiving OPS-CRM, IpaB (both at 10 μg/dose), AdjuPhos, or phosphate-buffered saline (PBS) were included as controls. Mice immunized intranasally (i.n.) with sublethal doses of S. flexneri 2a or S. sonnei were included as positive controls for challenge experiments. OPS-IpaB and OPS-CRM conjugates elicited high levels of OPS-specific serum IgG (Fig. 4A and B); titers were not significantly different between the two groups (Fig. 4B). As expected, high levels of OPS-specific serum IgG were detected in mice that received S. flexneri 2a i.n. (Fig. 4A). In all vaccinated groups, IgG titers increased with each vaccination and reached a plateau ~28 days after the boost (Fig. 4A and B). Mice immunized with S. flexneri 2a OPS-IpaB also produced high levels of IpaB-specific IgG, and titers surpassed those elicited by IpaB alone (Fig. 4A and B): mean values of 2.1 × 10^6^ and 1.4 × 10^6^ enzyme-linked immunosorbent assay (ELISA) units (EU)/mL, respectively, on day 55 postvaccination. High levels of CRM-specific serum IgG were also produced by OPS-CRM recipients (Fig. 4A). Importantly, S. flexneri 2a OPS-IpaB and OPS-CRM elicited antibodies that mediated serum bactericidal activity (SBA) against S. flexneri 2a; SBA titers were >10-fold higher than baseline in both groups (Fig. 4C). In contrast, no SBA activity was detected against S. sonnei Moseley and 53G strains (Fig. S2A). Sera from mice immunized with IpaB alone also lacked bactericidal activity (Fig. S2B). These results are consistent with the notion that OPS-specific antibodies are the main contributors to bactericidal activity and the recognized serotype specificity of OPS-induced immunity. In agreement, mice immunized with sublethal doses of S. flexneri 2a or S. sonnei Moseley displayed robust SBA only against the respective immunizing serotype, S. flexneri 2a or S. sonnei (Moseley and 53G) strains (Fig. S2).

10.1128/msphere.00019-23.2 FIG S2 Serum bactericidal activity (SBA) of OPS-induced antibodies against heterologous *Shigella* strains and lack of SBA of IpaB-specific antibodies. (A) Pooled sera from mice immunized with S. flexneri 2a OPS-IpaB or AdjuPhos (negative control) were tested for SBA against S. sonnei. Pooled sera from mice repeatedly infected with sublethal doses of S. sonnei were included as a positive control. Vaccinations are described in the legend to Fig. 4. (B) Pooled sera from mice immunized with IpaB and AdjuPhos (negative control) were tested for SBA against S. flexneri 2a. Pooled sera from mice infected with sublethal doses of S. flexneri 2a were included as a positive control. SBA assays were performed in triplicates. Mean of individual titers ± standard error of the mean; *, *P* < 0.0001 compared to AdjuPhos by *t* test. Download FIG S2, TIF file, 1.0 MB. Copyright © 2023 Desalegn et al. 2023 Desalegn et al. https://creativecommons.org/licenses/by/4.0/ This content is distributed under the terms of the Creative Commons Attribution 4.0 International license.

### S. flexneri 2a OPS-IpaB affords high levels of protection against S. flexneri 2a and S. sonnei lethal infection.

**FIG 5:**
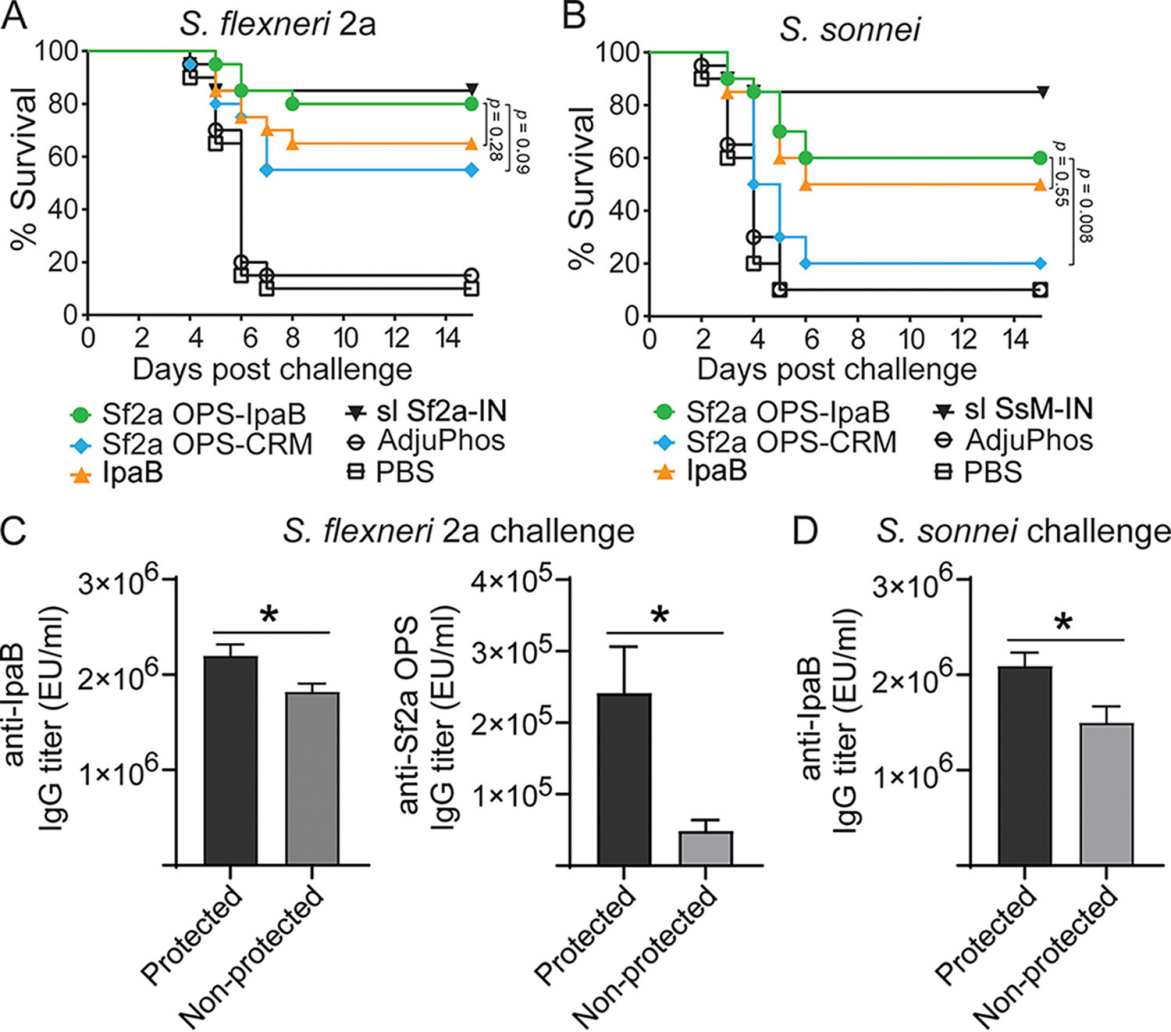
Protective efficacy of S. flexneri 2a OPS-IpaB conjugate vaccine. (A and B) Mice were immunized, as described in the Fig. 4 legend, and challenged i.n. with a lethal dose (5 × 10^6^ CFU) of S. flexneri 2a (A) or S. sonnei (B) on day 57 postvaccination. Data represent survival curves from 20 mice/group. *P* values determined by log rank (Mantel-Cox) test. (C and D) Comparison of vaccine-induced prechallenge IpaB- or S. flexneri 2a OPS-specific serum IgG titers between protected and nonprotected mice after S. flexneri 2a (C) and S. sonnei (D) challenges. Mean of individual titers ± standard error of the mean; *, *P* < 0.05 by *t* test. sl Sf2a-IN, sublethal dose of S. flexneri 2a-IN; sl SsM-IN, sublethal dose of S. sonnei Moseley-IN.

All groups were challenged i.n. (pulmonary infection model) with virulent S. flexneri 2a 2457T or S. sonnei Moseley on day 57 postvaccination. Immunization with S. flexneri 2a OPS-IpaB conjugate vaccine afforded 78% protection against homologous S. flexneri 2a challenge (*P* < 0.0001) whereas S. flexneri *2a* OPS-CRM provided only 50% protection (*P* = 0.0014) (Fig. 5A). IpaB alone conferred 67% protection against S. flexneri 2a (*P* = 0.0003) (Fig. 5A). The trend of higher protective efficacy of OPS-IpaB than of OPS-CRM or IpaB alone suggests that both OPS and IpaB contribute to the observed protective immunity. IpaB- and OPS-specific IgG titers in mice that were protected were significantly higher than titers in those that succumbed to infection (Fig. 5C).

Importantly, S. flexneri 2a OPS-IpaB exhibited 56% protection against heterologous S. sonnei challenge (*P* < 0.0001) (Fig. 5B). Because *Shigella* O-polysaccharide immunity is serotype specific, this cross protection is attributable to IpaB. This is consistent with the lack of protection in the OPS-CRM group (11% survival). IpaB alone afforded 44% protection against S. sonnei (*P* = 0.0003) (Fig. 5B), which was not significantly different from the protection elicited by S. flexneri 2a OPS-IpaB. IpaB-specific serum IgG was again significantly higher in mice that were protected against S. sonnei infection (Fig. 5D). S. flexneri 2a and S. sonnei IpaBs share >98% homology; therefore, cross protection was expected. The slight difference in IpaB efficacy in the two experiments is likely due to the higher severity of S. sonnei infection (mice succumbed sooner). Unvaccinated control mice had very low to no survival. A summary of the protective vaccine efficacy data is shown in Table 1.

**TABLE 1:** Vaccine efficacy against S. flexneri 2a and S. sonnei lethal infection in mice

S. flexneri 2a			S. sonnei		
Vaccine^*c*^	% VE^*a*^	*P* value^*b*^	Vaccine^*c*^	% VE^*a*^	*P* value^*b*^
OPS-IpaB	77.8	<0.0001	OPS-IpaB	55.6	<0.0001
OPS-CRM	50.0	0.0014	OPS-CRM	11.1	0.0405
IpaB	66.7	0.0003	IpaB	44.4	0.0003
sl Sf2a-IN	83.3	<0.0001	sl SsM-IN	83.3	<0.0001
AdjuPhos	5.6	0.5814	AdjuPhos	0	0.6346
PBS			PBS		

a % vaccine efficacy (VE) = [(% death in unvaccinated − % death in vaccinated)/% death in unvaccinated] × 100.

b Survival curves of vaccinated groups were compared to PBS control determined by log rank (Mantel-Cox) test. *P* values of <0.05 were considered statistically significant.

c sl Sf2a-IN, sublethal dose of S. flexneri 2a-IN; sl SsM-IN, sublethal dose of S. sonnei Moseley-IN.

## DISCUSSION

There is an urgent need for a well-tolerated, effective, and affordable vaccine to prevent *Shigella*-induced diarrhea. At-risk groups who will benefit the most include children (3 to 36 months of age) living in low- and middle-income countries, travelers to regions of endemicity, military personnel, the elderly, and immunocompromised individuals. We report here for the first time the successful development of a *Shigella* OPS-conjugate vaccine that employs IpaB as a carrier antigen. IpaB is known to be potently immunogenic (17, 18, 22, 26). IpaB-specific antibodies are present in high levels in sera from individuals living in areas of endemicity (27). Several groups have also reported the protective capacity of IpaB in animal models (17, 18, 28), and IpaB-specific antibodies (19, 20) and memory B cells (29) have been associated with reduced risk of shigellosis in humans.

The OPS-IpaB conjugate described in this study has several important advantages over existing *Shigella* conjugates: (i) the inclusion of IpaB results in more robust antibacterial immunity that targets the bacterial lipopolysaccharide as well as a virulence factor; (ii) immunity is no longer limited to the serotype-specific O antigen but now expands to a highly conserved antigen; (iii) it avoids the use of carrier proteins already included in several routine vaccines given to infants of the same age, which may interfere with vaccine responses. Additionally, two innovative features distinguish the production of OPS-IpaB from other *Shigella* conjugate vaccines: (i) efficient production of large amounts of nnAA-containing IpaB using the scalable Xpress^+^ CFPS platform—Vaxcyte has scaled CFPS to a 200-L-plus volume under good manufacturing practices (GMP) for production of other carrier proteins—and (ii) the site-specific conjugation of OPS to IpaB, which results in a more predictable and consistent product. IpaB has traditionally been produced in cell-based heterologous expression systems. However, this method has major limitations with regard to recovery and solubility (30–32). CFPS allows competent production of both native and nnAA-containing soluble IpaB at high yields and low cost. Unlike conventional methods that employ random conjugation, site-specific conjugation preserves functional and/or immunogenic epitopes.

S. flexneri 2a OPS-IpaB administered to mice i.m. in the presence of a traditional alum-based adjuvant was highly immunogenic and afforded high levels of protection against S. flexneri 2a lethal pulmonary infection. Mice that survived the lethal infection had higher levels of both OPS- and IpaB-specific IgG than did those that succumbed to infection, suggesting that both antigenic targets contributed to protection against S. flexneri 2a infection. The OPS-IpaB conjugate elicited OPS-specific antibodies that displayed functional (bactericidal) activity against S. flexneri 2a but not S. sonnei, which confirms the serotype-specific nature of OPS-induced immunity. Serotype-specific SBA responses have been reported in mice immunized i.m. with SF2a-TT15 (33), in rats that received S. flexneri 2a-rEPA (34), and in rabbits vaccinated with a monovalent and quadrivalent *Shigella* OPS-rEPA bioconjugate (35). The production of antibodies that can activate complement and mediate bacterial killing is important as *Shigella* SBA has been associated with reduced risk of S. flexneri 2a (19, 36) and S. sonnei (37) infection in humans. Further studies to elucidate the breadth of responses elicited by *Shigella* OPS-IpaB (duration, T cell responses, B memory, cytokine profiles, etc.) are warranted.

The S. flexneri 2a OPS-IpaB conjugate exhibited higher protective efficacy against homologous (S. flexneri 2a) lethal challenge than did OPS-CRM, which can be attributed to immunity against IpaB. This result suggests that a *Shigella* OPS-IpaB conjugate may provide protection superior to that of other current *Shigella* OPS conjugate vaccines, which use irrelevant proteins as carriers. Intriguingly, there are no reports in the literature describing the protective efficacy of S. flexneri 2a OPS-rEPA (Flexyn2a), S. sonnei OPS-rEPA, or SF2a-TT15 conjugate vaccines in animal models. Only antibody-mediated blocking of S. flexneri 2a infection in mice, not vaccine protection due to active immunization, was shown for SF2a-TT15 (33). Ours is the first demonstration of significant protective efficacy of a parenterally delivered *Shigella* conjugate vaccine in a lethal mouse infection model.

Importantly, the S. flexneri 2a OPS-IpaB conjugate also elicited robust cross-protective immunity against S. sonnei. The lack of protection in S. flexneri 2a OPS-CRM-vaccinated mice against a heterologous S. sonnei strain and the serotype-restricted SBA responses elicited by both OPS conjugates confirm that the cross protection afforded by the OPS-IpaB conjugate is dependent not on immunity to OPS but on that to IpaB. Another subunit *Shigella* vaccine that combines serotype-specific antigens with conserved proteins (IpaB and IpaC) is the Invaplex (38). Cross protection against heterologous serotypes of *Shigella* has been reported for the Invaplex vaccine in preclinical studies and attributed to the immune response stimulated by IpaB and IpaC (38, 39). Unlike sera from OPS-IpaB and OPS-CRM vaccine recipients, sera from mice that received IpaB alone did not exhibit SBA. These results confirm the OPS specificity of bactericidal humoral immunity. The exact mode of action of the IpaB-specific antibodies is not fully understood, although they are presumed to block *Shigella* host cell invasion. Recent system serology data produced by our group suggest that anti-IpaB antibodies may also facilitate phagocytosis by neutrophils and monocytes (20). Clearly, the inclusion of IpaB as carrier protein not only enhanced but also expanded the conjugate’s protective capacity (IpaB homology across *Shigella* species is >98%). The broader coverage of OPS-IpaB may also simplify the vaccine approach by circumventing the need for multiple conjugates to prevent illness caused by circulating serotypes. Further studies are ongoing to ascertain whether S. flexneri 2a OPS-IpaB or a bivalent S. flexneri 2a and S. sonnei OPS-IpaB conjugate vaccine formulation would be preferable to cover most prevalent *Shigella* serotypes.

Another advantage of the *Shigella* OPS-IpaB conjugate vaccine candidate is the use of a carrier protein (i.e., IpaB) that has not been used in other conjugate vaccines given to infants. Carrier proteins in existing *Shigella* OPS conjugates (e.g., TT and CRM_197_) are included in multiple other routine conjugate vaccines given to infants of the same age. Immunity to glycoconjugate carriers (preexistent or concurrently induced) has been shown to reduce responses to antigens coadministered with the same carriers. For instance, immunity to a TT-conjugate vaccine was reduced as a result of prior (40) or concomitant (41) immunization with a different TT-containing vaccine—this effect is known as carrier-induced immune suppression. In addition, coadministration of multiple CRM_197_-containing conjugate vaccines is at higher risk of inducing bystander interference than are schedules that do not include CRM_197_ (reviewed in references 42 and 43). The use of IpaB in our OPS glycoconjugate avoids these concerns, particularly as new conjugates are added to the infant vaccination schedules. The effectiveness of IpaB on its own, the breadth and features of IpaB immunity, and its properties as a carrier remain to be elucidated.

To the best of our knowledge, we are the first to report the development of a *Shigella* OPS-IpaB conjugate vaccine candidate and its ability to confer robust protective immunity against multiple *Shigella* serotypes. There are momentum in the field and strong recommendations for the advancement of *Shigella* vaccines (44). The OPS-IpaB glycoconjugate candidate described here meets the World Health Organization preferred product profile specifications for vaccines against *Shigella* (45) in terms of improved antigenic composition, expected safety based on the precedent of other conjugate vaccines, noninterference, parenteral delivery, and potential for robust immunogenicity and broad efficacy; its holds great potential for prevention of disease in the most vulnerable groups (i.e., infants and toddlers). Efforts to expand product analytical characterization and preclinical studies of immune responses and efficacy against other *Shigella* serotypes and clinically relevant strains with eventual evaluation in humans are ongoing.

## MATERIALS AND METHODS

### Production of *Shigella* core OPS.

S. flexneri 2a core OPS production was performed as previously described for purification of Salmonella enterica serovar Typhimurium core OPS (46), with modifications. Briefly, the core OPS was extracted from a live attenuated S. flexneri 2a strain (CVD1204) (21). The bacteria were grown in a chemically defined medium supplemented with 0.13 g/L amino acids, 0.025% guanine, and 50 μg/mL kanamycin to stationary phase, and core OPS was extracted by boiling the fermentation culture in ~6% acetic acid, pH 3.7, at 100°C for 4 h. Cellular debris was removed by centrifugation at 10,000 × *g*, 4°C, for 30 min, and the supernatant was brought to 1 M NaCl and clarified using a 0.2-μm hollow-fiber tangential flow filtration (TFF) membrane (Sartorius, Germany). The OPS in the filtered supernatant was concentrated over a 30-kDa Hydrosart TFF membrane and diafiltered 35-fold with 1 M NaCl to remove impurities, followed by 10-fold diafiltration with 20 mM Tris, 50 mM NaCl, pH 7, for buffer exchange into appropriate buffer for anion-exchange chromatography. The concentrated, buffer-exchanged OPS was passed over a Sartobind Q membrane (Sartorius, Germany) in negative mode to remove impurities, and the OPS was collected in the flowthrough. The OPS was then brought to 25% ammonium sulfate and incubated at 4°C for ~20 h. Precipitate was removed by centrifugation at 10,000 × *g*, 4°C, 30 min, followed by 0.45-μm filtration. The core OPS in the filtered supernatant was concentrated using a 10-kDa Hydrosart TFF membrane and diafiltered 10-fold into water. The core OPS was sterile filtered with a 0.2-μm vacuum filter, frozen, and lyophilized for dry-weight analysis. Identity for sugar moieties in the purified OPS was confirmed by high-performance anion-exchange chromatography with pulsed amperometric detection (HPAEC-PAD) with an ICS-6000 system and CarboPac PA10 column run at 0.35 mL/min using commercially available monosaccharide standards.

### Cloning, expression, and purification of nnAA-containing variant of IpaB.

The genes for expression of IpgC (amino acids [aa] 1 to 155, GenBank accession number AAP78992.1) and native or pAMF-containing IpaB (aa 1 to 580, GenBank accession number SVH88885.1) from S. flexneri 2a were synthesized at ATUM (Menlo Park, CA) and subcloned with an N-terminal methionine into a proprietary vector using NdeI and SalI as restriction sites. Each of the genes contained a C-terminal His_6_ tag for purification of the protein. *In vitro* native and pAMF-containing protein expression and titer estimation using the XpressCF^+^ CFPS platform were performed as described elsewhere (22). For purification of IpaB-variant 1 (expressed in the presence of exogenously added chaperone IpgC at an 0.3-mg/mL concentration), the process was scaled up at a 2- to 4-L reaction volume and controlled CFPS in a bioreactor was performed using a DASbox at 25°C for 10 h with constant stirring at 650 rpm while maintaining the pH at 7.2 and sparging with a blend of air and oxygen through the reaction mixture to maintain the dissolved oxygen at 30%. After 10 h, the reaction mixtures were harvested and spun down at 15,000 × *g* at 4°C for 30 min followed by filtration using an 0.45-μm pore size membrane. Thereafter, the clarified filtrate was loaded onto a 5-mL HisTrap Excel column preequilibrated with buffer A1 (50 mM Tris, 150 mM M NaCl, 10 mM imidazole, 0.1% lauryldimethylamine oxide [LDAO]), which in turn also helped remove coeluting IpgC from IpaB. Finally, the bound protein was eluted using a 50% step gradient of buffer A1 with 500 mM imidazole. Postcapture, the elution fractions for IpaB were combined, concentrated using 30-kDa-cutoff Amicon Ultra-15 centrifugal filters (Millipore Sigma, USA), and loaded onto a gel filtration Superdex200 26/60 column preequilibrated with buffer S1 (50 mM Tris, 150 mM M NaCl, 0.1% LDAO). Fractions with the highest purity (>95% as assessed by SimplyBlue SafeStain [ThermoFisher, USA]) after SDS-PAGE analysis were combined, aliquoted, and stored at −80°C for further use. Protein concentration was measured by *A*_280_ while subtracting the background absorbance for the buffer alone. Purified IpaB-var1 was incubated with excess DBCO-TAMRA dye for 1 h at room temperature to confirm pAMF incorporation. SDS-PAGE gel and fluorescence readout was recorded using a Syngene G-box gel imager.

### DBCO derivatization of OPS and conjugation to IpaB-var1 for generating conjugate vaccine.

The purified OPS resuspended to 6 mM in 100 mM borate buffer (pH 8.5) was combined with 3 equivalents (to the polysaccharide repeating unit [PSRU]) of 1-cyano-4-dimethylaminopyridinium tetrafluoroborate (CDAP; from 100-mg/mL solution in acetonitrile) with vigorous stirring to facilitate cyanylation at reactive hydroxyl groups. At 5 min after the addition of CDAP, 2 molar equivalents of dibenzocyclooctyne-amine linker stock in dimethyl sulfoxide (DMSO) was added to reach a final DMSO concentration of 5% (vol/vol). After DBCO derivatization, unreacted cyanate esters were quenched by addition of 200 mM glycine. The DBCO-derivatized OPS (OPS-DBCO) was purified via a Zeba spin column, and the purity of the material was assessed by reverse phase. A single peak in high-performance liquid chromatography (HPLC) when absorbance was monitored at 309 nm confirmed the complete removal of excess DBCO linker and other reaction by-products. Finally, the OPS concentration was measured using an anthrone assay, and dibenzocyclooctyne concentration was measured using absorbance at 309 nm. These two values were combined to give an estimate of the percentage of polysaccharide derivatized with a dibenzocyclooctyne functional group. For conjugation, the percent DBCO derivatization of the mutant polysaccharide (PS) was kept between 5 and 10%. Thereafter, IpaB-var1 was mixed with OPS-DBCO at a 1:1 ratio (0.5 mg/mL of each) to facilitate conjugation via click-chemistry reaction at room temperature. Postreaction, the reaction mixture was dialyzed against a 50-kDa-cutoff membrane to remove excess unreacted OPS-DBCO. The recovered conjugates were analyzed by SEC-MALS, and the final concentration of OPS and proteins in the purified conjugates were measured using anthrone and bicinchoninic acid (BCA) (Pierce protein assay kit; Thermo Scientific) assays, respectively. Both OPS and IpaB/CRM were present at a 1:1 ratio in the final purified conjugates; the 10-μg immunizing doses contained 10 μg of OPS conjugated to 10 μg of protein.

### SEC-MALS analysis.

The SEC-MALS UV-RI setup consists of an Agilent HPLC 1100 degasser, temperature-controlled autosampler (4°C), column compartment (25°C), and UV-visible (UV-Vis) diode array detector (Agilent, Santa Clara, CA) in line with a Dawn-Heleos multiangle laser light scattering detector and Optilab T-rEX differential refractive interferometer (Wyatt Technology, Santa Barbara, CA). The system was coupled to a Superdex200 10/30 Increase column for IpaB-var1 protein. A mobile phase consisting of 0.2-μm-filtered 50 mM Tris (pH 8), 150 mM NaCl, 0.1% (vol/vol) LDAO was used at a 0.5-mL/min flow rate. Approximately 50 to 100 μg of sample was injected for analysis. Agilent Open Lab software was used to control the HPLC, and Wyatt Astra 7 software (Wyatt Technology Corp., Santa Barbara, CA) was used for data collection and molecular weight analysis.

### Anthrone assay for estimating total polysaccharide concentration.

A stock of 2 mg/mL of the anthrone reagent (Sigma-Aldrich, St. Louis, MO; CAS number 90-44-8) was prepared in cold sulfuric acid while a 1 mM stock of polysaccharide repeating unit (PSRU) was prepared in water as a standard. In triplicate wells, 100 mL of PSRU stock (serially diluted into reference standards) or the unknown samples (diluted 1:3) was plated (96-well plate) followed by addition of 200 mL/well of the anthrone reagent stock. All reaction mixtures were thoroughly mixed and sealed with a plate cover for incubation at 95°C for 10 min. The plate was briefly placed on ice to cool to ambient temperature before absorbance was measured at 620 nm using a UV-Vis plate reader. To determine concentration of unknown samples, PSRU standard concentrations and absorbances were used to generate a least-square fit regression.

### Ethics statement.

The current study was performed in accordance with guidelines from the “Guide for the care and use of laboratory animals” of the National Institutes of Health (NIH). All animal studies and procedures were approved by the University of Maryland School of Medicine Institutional Animal Care and Use Committee (IACUC). All relevant ethical regulations for animal testing and research were followed. All efforts were made to minimize pain and distress of the animals.

### Mice, immunizations, and challenge.

Adult female BALB/c mice (6 to 8 weeks old) were purchased from Charles River Laboratories, Wilmington, MA. Mice were immunized i.m. on days 0 and 28 with 10 μg S. flexneri 2a OPS-IpaB, S. flexneri 2a OPS-CRM, and IpaB adsorbed to AdjuPhos (4.8%, vol/vol; InvivoGen, CA). The OPS-IpaB or OPS-CRM doses contained 10 μg of each antigen in the conjugate. i.m. immunization was performed by dispensing a 100-μL volume (50 μL per leg). Negative-control mice received i.m. AdjuPhos or PBS. Positive-control groups were immunized i.n. on days 0 and 28 with sublethal doses (~1 × 10^5^ CFU) of S. flexneri 2a 2457T or S. sonnei Moseley for respective challenge strains. At 4 weeks after the last immunization, mice were challenged i.n. with ~5 × 10^6^ CFU of S. flexneri 2a 2457T or S. sonnei Moseley (corresponding to ~4 or ~2 50% lethal doses [LD_50_], respectively). S. flexneri 2a and S. sonnei inoculum preparation and i.n. challenge were performed as previously described (17). Mice were monitored daily for 15 days after the challenge, and their health status, weight, and survival were recorded. Mice that reached a moribund state or lost more than 20% of their initial body weight and did not recover within 72 h were humanely euthanized and considered nonsurvivors. Blood was collected at day −1 (prevaccination) and days 13, 27, 42, and 55 (postvaccination) for serum antibody measurement. Blood collection via retro-orbital or submandibular vein and i.n. infection were performed under isoflurane anesthesia (VetEquip, Inc., CA).

### Antibody measurement.

Mouse IpaB-, S. flexneri 2a OPS-, and CRM-specific serum antibodies were measured by enzyme-linked immunosorbent assay (ELISA) as previously described (17, 22). For measurement of conjugate vaccine-induced OPS-specific antibodies, Nunc MaxiSorp plates (Thermo Scientific) were coated with S. flexneri 2a OPS-CRM (5 μg/mL) or OPS-IpaB (5 μg/mL) conjugates; for analysis of IpaB- or CRM-specific antibodies, Immulon 2HB plates (Thermo Scientific) were coated with IpaB (0.1 μg/mL) or CRM (5 μg/mL), respectively. Serum samples were tested in 2-fold serial dilution in duplicate. Horseradish peroxidase (HRP)-labeled goat anti-mouse IgG (KPL SeraCare, Gaithersburg, MD) was used as detection antibody. For evaluation of IpaB immune reactivity, plates were coated with IpaB or IpaB variants (produced by CFPS). A pool of human convalescent-phase sera was added and serially diluted 2-fold starting at 1:20,000. Bound antibodies were detected with HRP-goat anti-human IgG (Jackson Immuno Research, West Grove, PA). Both values of optical density at 450 nm (OD_450_) values and endpoint titers were obtained. Endpoint titers were calculated by interpolation of absorbance values of samples in the linear regression curve of a calibrated in-house standard and were reported as ELISA units (EU)/mL. An ELISA unit corresponds to the reciprocal serum dilution resulting in an absorbance value of 0.2 at 450 nm above the background.

### SBA.

The serum bactericidal activity (SBA) assay was conducted as previously described (47), with modifications. Briefly, heat-inactivated mouse sera were serially diluted 3-fold in assay buffer in 96-well U-bottom plates (Fisher Scientific). S. flexneri 2a 2457T or S. sonnei Moseley and 53G strains (~120 CFU in 10 μL) and 50 μL of 20% baby rabbit complement (BRC) (Pel-Freez Biologicals, AR) were then added to the wells. Plates were incubated for 2 h at 37°C without shaking. Negative controls contained the bacteria and BRC only. A standard with assigned SBA titer was included in each assay and used for titer normalization. Colony counts were enumerated using NIST Integrated Colony Enumerator (NICE) software, and an Excel-based software program, Opsotiter, was used to determine SBA titers.

### Statistical analysis.

Statistical analyses were conducted using GraphPad Prism 9 (GraphPad Software, La Jolla, CA). Antibody titers were analyzed by unpaired *t* test. Survival curves of vaccinated and control groups were analyzed and compared using a log rank (Mantel-Cox) test. *P* values of <0.05 were considered statistically significant.
