# Supplementary figures and images for: A Novel Shigella O-Polysaccharide–IpaB Conjugate Vaccine Elicits Robust Antibody Responses and Confers Protection against Multiple Shigella Serotypes
Source: mSphere. 2023 Apr 5;8(3):e00019-23. doi: 10.1128/msphere.00019-23 (PMC10286710; doi:10.1128/msphere.00019-23)

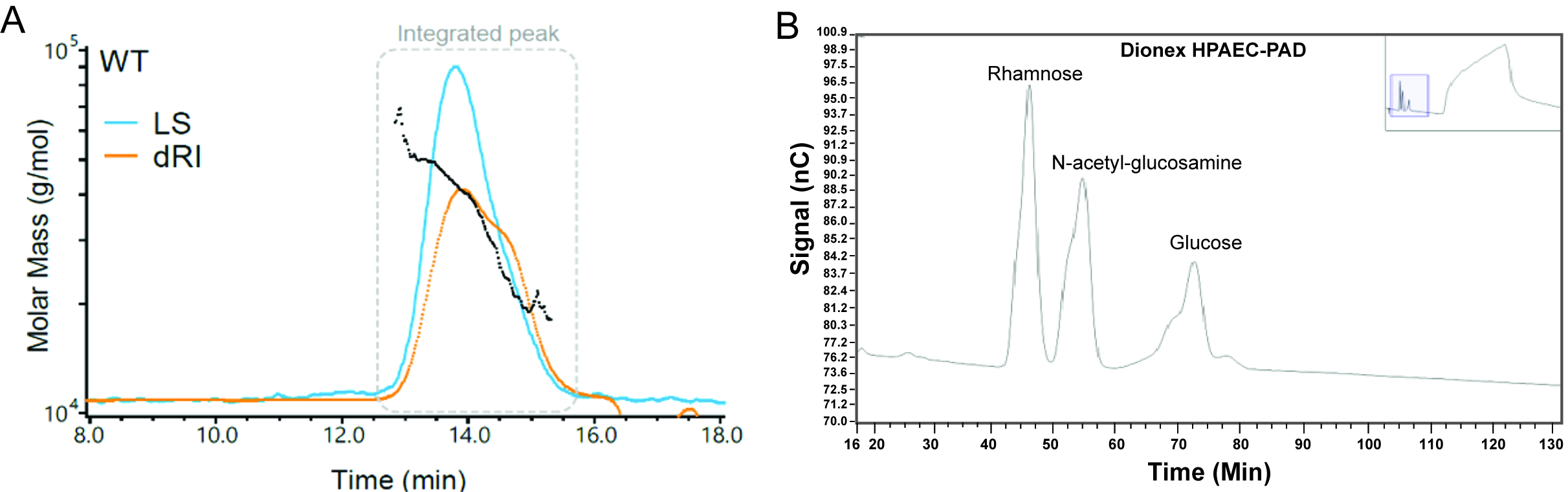

Supplement: FIG S1 [file msphere.00019-23-s0001.tif]

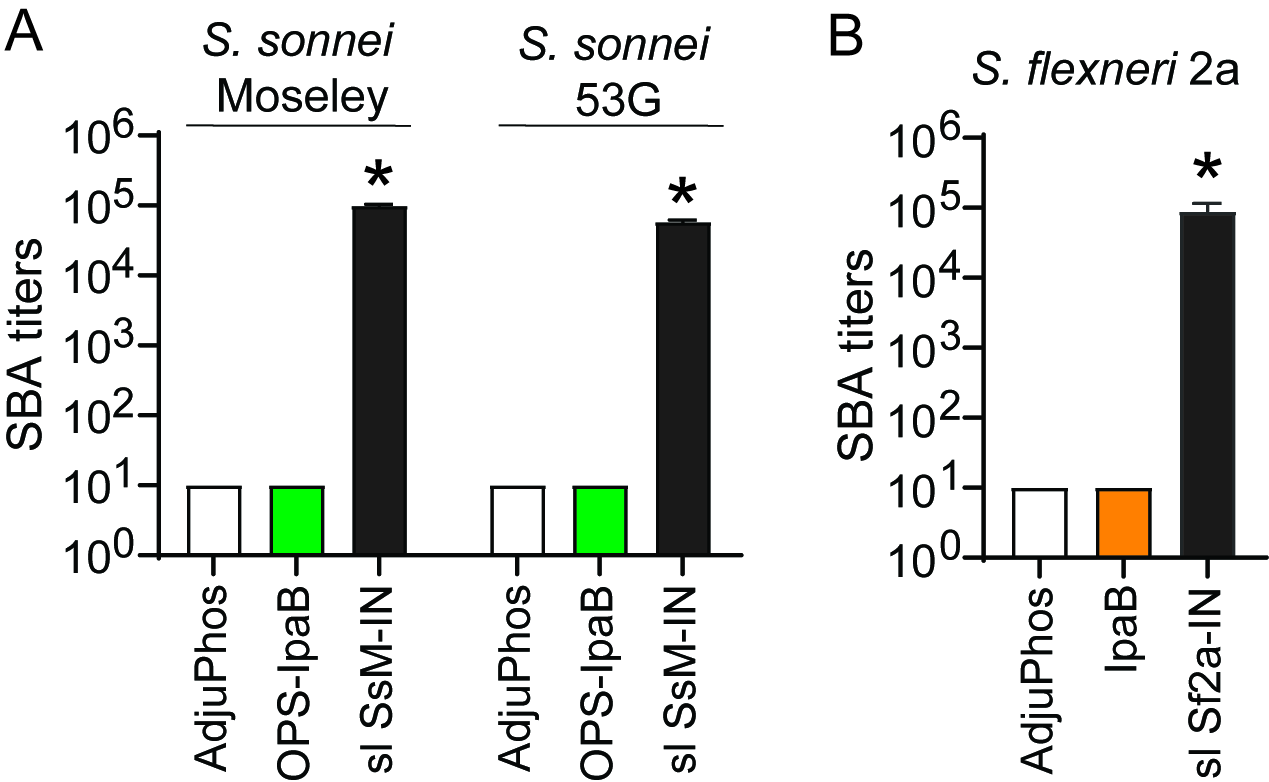

Supplement: FIG S2 [file msphere.00019-23-s0002.tif]
